# Image-based prediction of residential building attributes with deep learning

**DOI:** 10.1111/jiec.13591

**Published:** 2024-11-19

**Authors:** Weimin Huang, Alexander W. Olson, Elias B. Khalil, Shoshanna Saxe

**Affiliations:** 1 Department of Mechanical and Industrial Engineering, University of Toronto, Toronto, ON Canada; 2 Centre for Analytics and AI Engineering, University of Toronto, 55 St George Street, Toronto, ON M5S 0C9 Canada; 3 Department of Civil and Mineral Engineering, University of Toronto, Toronto, ON Canada

**Keywords:** building attribute estimation, Google Street View, industrial ecology, machine learning, material stocks, urban sustainability

## Abstract

This study estimates building attributes—floor area and age—using image-based machine learning. Building age and floor area are key inputs to the studies of urban metabolism, material stocks and flows, and embodied greenhouse gases (GHGs) in the built environment. However, these data are challenging to generate and maintain using traditional survey methods, their availability is uneven and often, even when available, very uncertain. Improving our understanding and future management of built environment resource flows and associated environmental impacts requires more complete access to building age and floor area data. The study formulates area prediction as a regression problem and age prediction as a classification problem over six historical periods, achieving a mean absolute percentage error of 19.42% for area prediction and an accuracy of 70.27% for age prediction in Toronto. These results are obtained using an EfficientNetV2 module for feature extraction from Google Street View images, followed by fully connected layers for estimating the two building attributes. The performance of the Toronto-trained model in five other Canadian cities is also reported, highlighting the model's varying effectiveness in different urban contexts and the benefit of local training. Our findings demonstrate the feasibility of using machine learning for building attribute estimation from street-view images, offering a basis for future automated large-scale material flow and stock analysis.

**Supplementary Information:**

The online version of this article (doi:10.1111/jiec.13591) contains supplementary material, which is available to authorized users.

## INTRODUCTION

In this research, we use image-based machine learning to estimate two attributes of buildings—floor area and age—both key metrics in the study of material flow and stock analysis, as well as urban metabolism and embodied GHG assessments. Population growth and changes in family formation have driven demand for new buildings and upgraded housing over the past century. In parallel, the material stock in the built environment has substantially increased and is still accelerating in much of the world (Krausmann et al., 2017; Zhong et al., 2021). The resulting resource demands and environmental impacts associated with construction materials use increasingly diverge from what is consistent with a sustainable future (Zhong et al., 2021).

The growing volumes of construction materials accumulated in buildings represent a reservoir of secondary raw materials and have the potential for reuse and recycling (Lanau et al., 2019). Understanding the quantity and location of embodied materials in buildings can help identify reusable components and available secondary resources. In addition, floor area assessment can help identify building space where the utilization rate could be improved which would reduce the need for new buildings. Maintaining information on building attributes such as the floor area and age can help support the design of more sustainable buildings (e.g., more efficiently designed residential units; United Nations Environment Programme, International Resource Panel, 2020).

With the growth in research on applying machine learning (ML) and artificial intelligence (AI) to sustainability and problems of societal relevance (Berendt, 2019; De-Arteaga et al., 2018; Dietterich, 2009; Gomes et al., 2019; Gomes, 2009; Hager et al., 2019; Rolnick et al., 2022), there is also increasing interest in utilizing AI and ML to understand the built environment more efficiently. Our research aims to use ML to predict the floor area and age of residential buildings from Google Street View (GSV) images to facilitate large-scale bottom-up flow and stock analysis, embodied GHG assessment, and urban metabolism calculations. While recent research has demonstrated the suitability of deep learning for age prediction in buildings (we compare our results to those in the literature in Section 4), to our knowledge there is no research applying image-based neural networks to the task of predicting building internal area. We use an EfficientNetV2 module to extract image features from GSV images, followed by fully connected layers for building attribute estimation. Area and age prediction are formulated as regression and classification problems, respectively. Our model achieves a mean absolute percentage error (MAPE) of 19.42% on area prediction. On age prediction, with six possible classes, our model achieves an accuracy of 70.27%. We also report model performance (without retraining) on five other Canadian cities: Calgary, Hamilton, Moncton, Montreal, and Victoria.

While internal floor area is not directly viewable from external street-view imagery (SVI), it is expected to be proportional to the external size of the building and is the kind of measure an informed human (e.g., architect, engineer, planner, someone shopping for a home, and so on) would be able to guess well. It is our hypothesis that information about the external size of the building, combined with available information including architectural style, number of windows, and the building's urban context, is sufficient for a useful estimate of the internal square footage and/or age to be produced.

Our approach expands upon existing utilization of artificial intelligence to automatically analyze the built environment by moving from prediction of externally visible attributes such as the age of a building, to include internal attributes, such as internal floor area that cannot be directly observed. In this way we seek to demonstrate the value of artificial intelligence to predict attributes only indirectly linked to the data being used.

## BACKGROUND

This section begins by reviewing recent machine learning contributions to material stock analysis. We then present recent papers that attempt to solve comparable tasks to our own. While there is significant literature on predicting the age of buildings, we did not identify papers that discuss predicting the internal area of a building from external features. The literature on these tasks is summarized in Table Table 1. Finally, we discuss EfficientNet, establishing the context for its development and our motivation for selecting it.

**TABLE 1:** Existing work on machine learning and artificial intelligence applied to building age prediction.

Reference	Task	Method	Input Data	Region	Results
Zeppelzauer et al. (2018)	Age (classification): 6 classes from 1960s to 2010s	SIFT, K-Means, CNN	Images from real estate valuation reports and web images	Austria	Renovated houses were excluded from training. The classification accuracy is 61% on a test set with no renovated buildings but drops to 35% when renovated buildings are included
Sun et al. (2022)	Age (classification): 9 classes from pre-1652 to 1995-2020	CNN	Street-view images	Amsterdam	Accuracy is 81% but drops to 24% when the model trained on buildings in Amsterdam is applied to buildings in Stockholm
Li et al. (2018)	Age (regression)	CNN and support vector regression	Street-view images	Victoria, Australia	The MAE and RMSE are 11 and 12 years, respectively
Lee et al. (2015)	Age (classification): 10 classes from pre-1800 to post-2000	Mid-level visual features representation, discriminative elements discovery	Street-view images	Paris	Many patches discovered capture key elements known to be prevalent in their respective periods

### Image-based building data prediction

A growing body of literature applies machine learning techniques to address challenges in automated analysis from images of buildings.

One approach demonstrated by Bao et al. (2023) leverages deep learning for grid-level estimation of material stock from high-resolution satellite images and nighttime lights (NTL) data. This method involves dividing satellite imagery into grids and using a ResNet-18 model to extract features from each grid. These features are then concatenated with spatiotemporal NTL features to perform regression, offering a novel perspective in material stock quantification.

Similarly, Yuan et al. (2023) focus on predicting the material stock of individual buildings in Hong Kong. This is achieved through multiple linear regression models that utilize a range of numerical and categorical building attributes, such as floor height, total height, and perimeter–floor area ratio. These attributes are categorized into building type, year, height, perimeter, floor area, and number of storeys, with some directly sourced from databases and others derived, for instance, counting storeys using Google Earth 3D views.

NTL data has also been utilized for its strong correlation with human activities and socioeconomic parameters. Studies have assumed a linear relationship between NTL radiance and material stock, employing linear regression for estimation. However, this approach has limitations, including potential inaccuracies due to full NTL saturation and background noise. As an alternative, Peled and Fishman (2021) estimate building volume based on NTL radiance, subsequently calculating stock using material intensity data. Despite these advances, challenges persist in capturing nonluminous structures and in the limited spatial resolution of NTL data, which can hinder the identification of material composition and building types.

Another promising technique is facade segmentation, as explored by Dai et al. (2021) and Arbabi et al. (2022). Facade segmentation models yield details like the number of storeys and total window area, significantly contributing to building energy modeling and material stock analysis. For instance, Arbabi et al. (2022) estimate the number of components like doors and windows in buildings through facade segmentation. This method also aids in building height estimation by facilitating camera location calibration, an essential element in height estimation methods based on camera projection models.

### Building age estimation

Building age plays an essential role in architectural history, building energy modeling, real estate valuation, and urban planning. Construction age data is often not available (Sun et al., 2022). Zeppelzauer et al. (2018) propose a framework to automatically classify building images into six decades. In their framework, they select representative patches (i.e., areas cropped from images), perform patch-level age classification, and then fuse the patch-level prediction results to obtain an image-level (i.e., building-level) prediction. They use convolutional neural network (CNN) models Alexnet (Krizhevsky et al., 2017) and ResNet-50 (He et al., 2016) to classify the patches into six classes. Importantly, they hypothesize that the existence of renovated houses makes it more difficult to estimate the year built; therefore, they excluded renovated houses from the training set. Their classification accuracy is 61.35% on a test set with no renovated buildings but drops to 34.94% when evaluated on another test set that includes renovated buildings. Sun et al. (2022) use deep learning to classify images of buildings obtained from GSV in Amsterdam, the Netherlands into nine age classes from early stages (pre-1652), to the contemporary era (1978–1994, 1995–2020). They achieve an accuracy of 81.09%. Instead of performing a patch-level classification, they directly classify the age of the buildings from the input images using a DenseNet121 model (Huang et al., 2017). To address the problem of buildings being too far away or being occluded in an image, they use a segmentation model to calculate the percentage of buildings in the image and only keep images where (1) the percentage of building categories is the highest among all categories and (2) buildings occupy more than 40% of the image. However, the accuracy of the segmentation model on the selected dataset was not verified. Similarly, Meng et al. (2022) classify images of building facades extracted from oblique aerial images in villages in Tianjin, China into three age classes: pre-1949, 1949–1980, and post-1980 and achieve an accuracy of 88.0%.

While the aforementioned studies (Meng et al., 2022; Sun et al., 2022; Zeppelzauer et al., 2018) formulate age prediction as a classification problem, Li et al. (2018) formulate it as a regression problem and apply a combination of deep learning and support vector regression (SVR) to estimate building age from GSV images in Victoria, Australia. They first use a pre-trained model to obtain image feature vectors from GSV images, and then feed the vectors into an SVR model to predict the year of construction. The mean absolute error (MAE) they obtained is 10.689 years.

A similar line of research is to identify architectural elements that are distinctive to particular construction periods. Singh et al. (2012) propose a framework for discovering a set of discriminative patches that can serve as a mid-level visual representation. Mid-level discriminative patches correspond to parts or objects that occur frequently and are different from the rest of the “visual world” so that they can capture the “essence” of that data. The main idea of their algorithm is to start with an initial clustering of the patches and perform iterative discriminative clustering. Lee et al. (2015) adapt this algorithm to identify visual elements distinctive to 10 construction periods in Paris from GSV images.

### EfficientNet

EfficientNet (Tan & Le, 2019, 2021) is a family of convolutional network models developed to address scaling challenges in deep neural networks. Historically, the scale of a neural network model has been primarily dictated by the amount of resources available, primarily in terms of computing power and training data. With more resources, developers generally opt to use a bigger network. However, making a neural network “bigger” was not a well-defined task until recently: “bigger” could mean more layers, more neurons in each layer, or an increase in many other aspects of the model. EfficientNet sought to standardize this scaling by introducing a single coefficient, which is then used to calculate suitable values for all these scalable parameters. In this way, the approach replaces a naive attitude to scaling with a more rigorous and, crucially, testable definition.

The efficacy of this approach has been demonstrated repeatedly in a range of image-based tasks. For the CIFAR-10 image classification benchmark, in which the model must correctly classify images into 1 of the 10 categories, the current state-of-the-art method correctly categorizes 99.9% of images using a network with 632 million parameters (Dosovitskiy et al., 2020). An EfficientNet approach correctly categorizes 99.1% of images using a network with 121 million parameters (Tan & Le, 2021) for the same task. Similarly, state-of-the-art performance on the ImageNet classification benchmark is reported at 91.0% correct using a model with 2.1 billion parameters. The best-performing EfficientNet approach achieves 90.2% correct with 480 million parameters (Pham et al., 2021). EfficientNet has been shown to be effective in the related task of estimating building height from GSV images (Olson and Saxe, 2024).

In summary, while substantially larger models can outperform EfficientNet-based approaches, EfficientNet offers the ability to intelligently scale a CNN to the resources available, reducing the risk of overfitting (Tan & Le, 2019) and greatly speeding up training. We selected EfficientNet for this research based on the risk of overfitting (and associated negative consequences for generalization) to the relatively small dataset gatherable in a given city/region for these tasks (e.g., tens of thousands of images rather than millions of data points).

## METHODS

### Data

To develop our models, images of buildings and the ground truth (age and area of buildings) are needed. The ground-truth data used in the project is obtained from a database of property appraisals provided by Real Property Solutions (RPS) Canada. RPS gathers information from the Municipal Property Assessment Corporation (MPAC) that assesses the value of properties according to property attributes (e.g., square footage, lot size, and age of home). To conduct assessments, MPAC reviews information on open market transactions between buyers and sellers (How sales affect your property assessment, n.d.). The RPS database includes cleaned and standardized records of approximately 8 million transactions across Canada beginning in 2004, covering more than 5 million properties. A wide array of information, including the location and a detailed description of the property, is provided for each transaction in the RPS database, including our variables of interest—street addresses, age, and living area of the building. The living area is the total above-ground area of the inside of the building and does not include the area of the basement, deck, porch, and garage (How we assess residential properties, n.d.).

**FIGURE 1:**
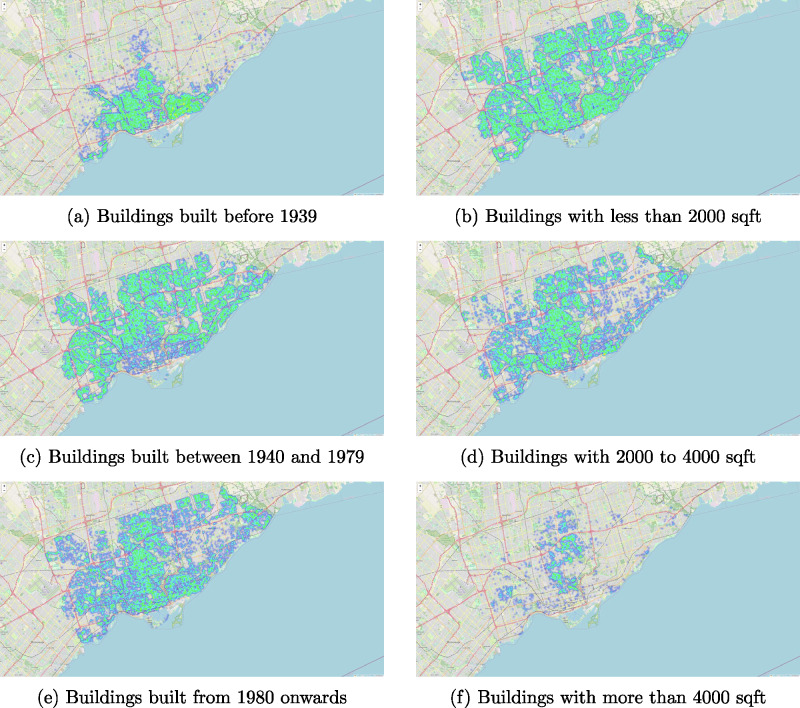
Heatmaps of buildings in Toronto by year built and square footage.

We preprocessed the transaction data obtained from the RPS database. Records that had missing information for our variables of interest were filtered out. Also, only the most recent transaction is selected when multiple transactions have been conducted for the same property (e.g., if it has been sold multiple times). The age and area variables in the database are used as the ground truth for training and validating the models (Figure 1).

**FIGURE 2:**
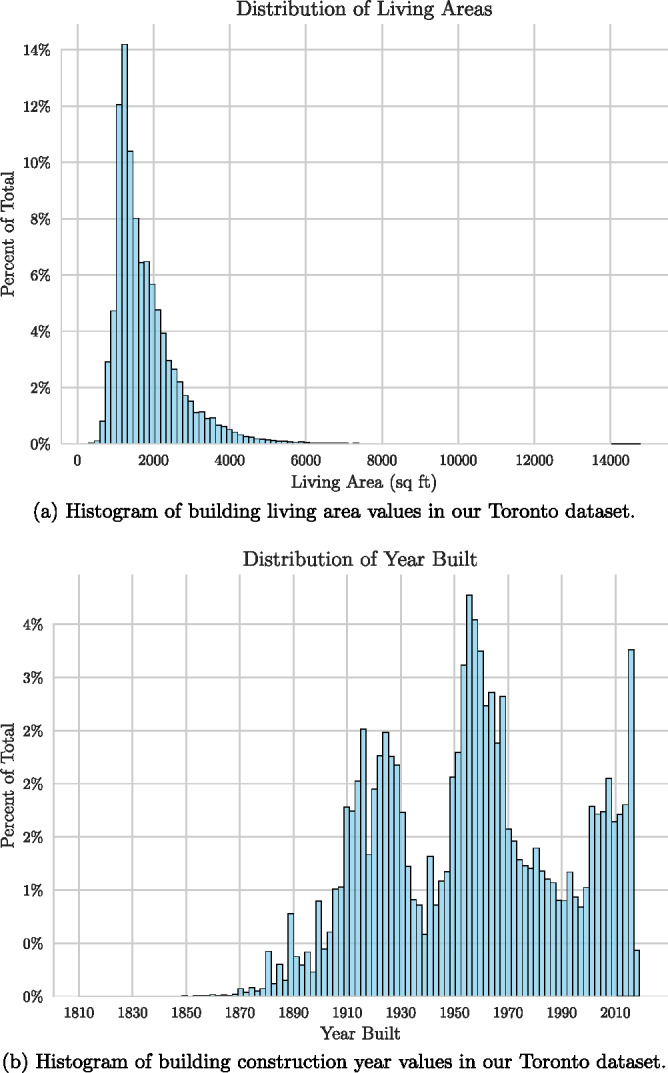
Histograms of building living area and building construction year values in our Toronto dataset. Underlying data are available in Table 1a and 1b of supporting information, respectively.

The building images used in the project are collected from the GSV Application Programming Interface (API) using the street addresses in the RPS database. For each building in the database, a request with the street address is passed to the API, and the returned image is downloaded. The size of the downloaded images is set to 640-by-640 pixels. The default horizontal field of view of the camera (90 degrees) is used.

For training and primary model evaluation, the City of Toronto was selected as it has the largest number of records in the dataset. From the Toronto data, 100,000 addresses were randomly selected. A total of 99,546 images were collected for Toronto (slightly less than the original 100,000—the Google Maps API geo-locates addresses to the correct location in the city, and was unable to return a precise location for these addresses). One image was captured per building. During the research, the use of multiple images per building was investigated but proved both difficult to acquire from the raw data and not to improve the accuracy of the model. The area and age distributions for buildings in the Toronto data are shown in Figures 2a and 2b, respectively. We also collected images from five other Canadian cities with large representation in the RPS dataset: Calgary, Alberta; Hamilton, Ontario; Moncton, New Brunswick; Montreal, Quebec; and Victoria, British Columbia (see Table 2). For each of these cities, we collected approximately 1000 images to be used for assessing whether the model, trained on Toronto data, could generalize to other cities. We also tested a ResNet-18-based model as a baseline and found the EfficientNet-based model performed better.

**TABLE 2:** General statistics of selected canadian cities (data from the 2021 Canadian Census).

City	Province	Population	Area ($\rm{km}^2$)	Distance to Toronto (km)
Toronto	Ontario	2,794,356	631	0
Calgary	Alberta	1,306,784	821	2710
Hamilton	Ontario	569,353	1118	59
Moncton	New Brunswick	79,470	141	1177
Montreal	Quebec	1,762,949	365	506
Victoria	British Columbia	91,867	19	3389

**FIGURE 3:**
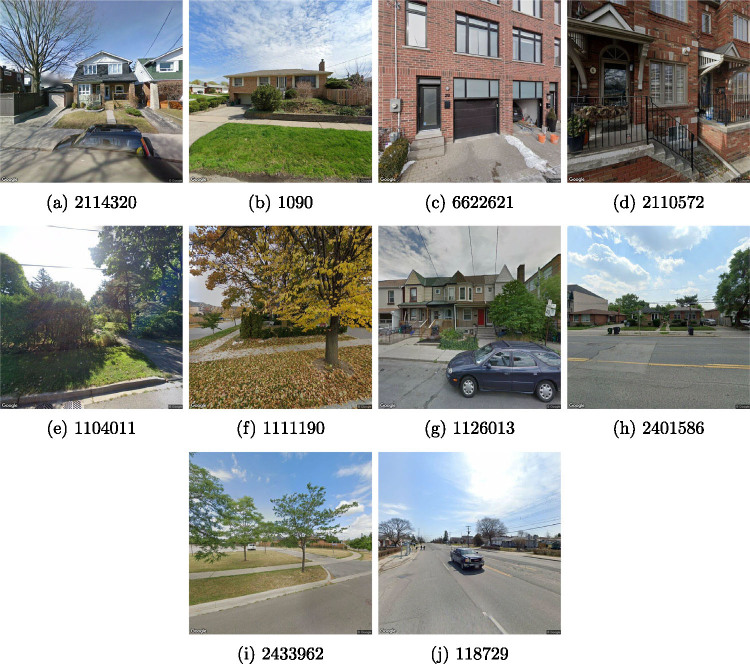
Example building images collected with Google Street View. The unique identifier of each image in our dataset is shown under it.

Example building images are shown in Figure 3. While the images collected with the Google API successfully capture the entire facade for some buildings (3a,b), there are cases where the buildings are occluded by trees (3e,f), where only a portion of the facade is visible (3c,d), or when the building is far from the camera and a small proportion of the pixels (3i,j). Moreover, more than one building is visible in the image when adjacent buildings are close together (3g,h). In these cases, it can be hard for humans to identify which one corresponds to the targeted building in the RPS database.

### Deep learning models

Using the data, we apply deep learning to predict the total floor area and age from the images of buildings. We use the EfficientNet-V2-S model for image feature extraction and then predict the attributes.

#### Image feature extraction

We utilize the EfficientNet-V2-S architecture (Tan & Le, 2021) to extract features from images for both area and age prediction tasks.

We initialize the EfficientNet-V2-S model using weights pre-trained on ImageNet (Krizhevsky et al., 2017), a large-scale dataset with over 15 million images in over 22,000 categories. This step is crucial to reduce overfitting and improve model performance. We then additionally pre-train the model on the Cityscapes dataset (Cordts et al., 2016), a large-scale database containing a diverse collection of annotated street scene images from 50 cities. This pre-training involves a modified task: predicting the percentage of the image that contains buildings. This step helps the model adapt better to urban imagery and enhances performance on our specific task.

Classification (used here for age prediction) and regression (used here for floor area prediction) tasks require inherently different metrics because classification measures discrete outcomes, best evaluated by accuracy, while regression predicts continuous values, best evaluated by MAPE (César Ferri et al., 2009).

#### Area prediction

Area prediction is formulated as a regression problem in which we predict a real number, the living area. In our dataset, the distribution of area is skewed; there is a small number of houses that have an area larger than 6000 square feet, with the largest samples in our dataset passing 14,000 square feet (Figure 1a). It is undesirable if a few houses with a very large floor area may dominate the average prediction error during training and evaluation. We reduce the impact of such outliers by minimizing a log-transformed version of the MAE, which is defined as:

1 $$ \rm{Log MAE}= \frac{1}{N} \sum _{i=1}^N {\left|\rm{log}(y_i)-\rm{log}(\bar{y_i})\right|}. $$

Here, $y_i$ is the target (ground-truth) value for the $i{\mathrm{th}}$ building in the training set and $\bar{y_i}$ is the predicted value for that same building. This loss function ensures that while the error for uncommonly large buildings is still considered, it is not vastly more important to the model than the far more common smaller buildings.

We consider the standard MAE (i.e., not log-transformed) during evaluation, as it is easier to understand. We also compute the MAPE, which is defined as the MAE divided by the target value for each instance. This allows us to directly compare the error between buildings of substantially different sizes. An absolute error of 20 m has a significantly different impact for a skyscraper than for a single family dwelling, for example. By considering the MAPE, we can more intuitively compare this relative difference in accuracy.

#### Age prediction

Age prediction is formulated as a classification problem. We group buildings into six classes: pre-1920, 1920–1940, 1940–1960, 1960–1980, 1980–2000, and post-2000. These 20-year periods cover the construction of nearly all standing buildings in Canada (e.g., in contrast to efforts in Europe with many older buildings and wider time bands). The model outputs a vector of six values corresponding to each of the classes. The values are in the interval [0,1] and can be interpreted as the likelihood (probability) of each class being the correct label. The class with the highest predicted value is taken as the prediction for that building. During training, we minimize cross-entropy (CE), a standard loss function for multi-class classification. CE is calculated as follows:

2 $$ \rm{CE} = -\frac{1}{N} \sum _{i=1}^{N} \sum _{j=1}^{6} y_{ij} \cdot \log \frac{\exp ({p_{ij}})}{\sum _{k=1}^C \exp ({p_{ik}})} $$

Here, $y_{ij}$ is the ground-truth label of building $i$ ($y_{ij}=1$ if building $i$ belongs to class $j$, and 0 otherwise), and $p_{ij}$ is the raw score that building $i$ belongs to class $j$ predicted by the model. This loss function ensures that the model is penalized for incorrect classifications and the degree of certainty with which it makes incorrect classifications.

In addition to CE, we compute the MAE as the distance between the correct index of the age class (a value between zero and five) and the predicted index. We also calculate the accuracy (fraction of buildings for which the correct age class was predicted), and balanced accuracy, which is the accuracy with equal weighting for instances from each class. Finally, we calculate the “off-by-one” accuracy, indicating the percentage of predictions within one class of the true value.

#### Image augmentation

Image augmentation is a crucial step in our model training process to enhance the diversity of our training dataset and reduce the risk of overfitting. We employ several augmentation techniques to simulate different imaging conditions and perspectives. These are random rotations (within 10 degrees of the original image), horizontal flips (mirroring), and color jitter (adjusting brightness, contrast, saturation, and hue). By transforming our images in these ways, we improve the robustness of our model to variations in real-world data.

Each image is randomly augmented during training, meaning that the model rarely sees the same image twice. Random augmentation encourages the model to learn more general features rather than memorizing specific images. For instance, a building's age or area should be identifiable regardless of whether the image is slightly darker or mirrored. Augmentations are not applied during validation and testing, so performance can be measured consistently at these stages.

### Implementation

The models are implemented using PyTorch in Python. We use 75% of the buildings in the dataset for training, 15% for validation, and 10% for testing. For each experiment, we utilized the Optuna hyperparameter optimization framework (Akiba et al., 2019). Hyperparameter tuning is essential to ensure optimal settings for many adjustable components of the deep learning pipeline. The following hyperparameters were tuned, with the corresponding ranges or options considered:

– Dropout rate: 0.0–0.5
– Learning rate (LR): $10^{-5}$–$10^{-1}$
– Weight decay: $$10^{-5}$$–$$10^{-1}$$
– Activation function: ReLU, SiLU, Mish
– Optimizer: Adam, AdamW, Lion

In both age and area prediction, the model is trained continually until three successive epochs of no improvement in validation loss are recorded. The model that achieved the lowest validation error for each task was selected and used as the final model for the evaluation on out-of-sample test data.

## RESULTS

For each task, in addition to our held-out test set of Toronto buildings, we test the model on images collected from five other Canadian cities. Testing on other cities was done to evaluate the model's capability to generalize to different architectural styles and understand to what extent additional training may be required to apply the model to new locations.

### Age prediction

The age prediction results on the Toronto test set and the other cities are displayed on the left in Table 3.

**TABLE 3:** Test performance for age and area prediction. MAE and MAPE refer to mean absolute error (MAE) and mean absolute percentage error (MAPE), respectively. All metrics are defined in Section 3.2. Lower values are better for MAE and MAPE; higher values are better for the other metrics.

	Age prediction				Area prediction	
City	MAE	Accuracy (%)	Balanced accuracy (%)	Off-by-one accuracy (%)	MAE	MAPE
Toronto	0.54	70	69	89	366	19.42%
Calgary	0.53	66	48	90	329	19.08%
Hamilton	1.46	40	48	62	308	19.45%
Moncton	0.83	46	43	81	417	23.10%
Montreal	1.46	36	38	60	644	27.12%
Victoria	1.05	41	39	72	626	34.67%

#### Toronto results

**FIGURE 4:**
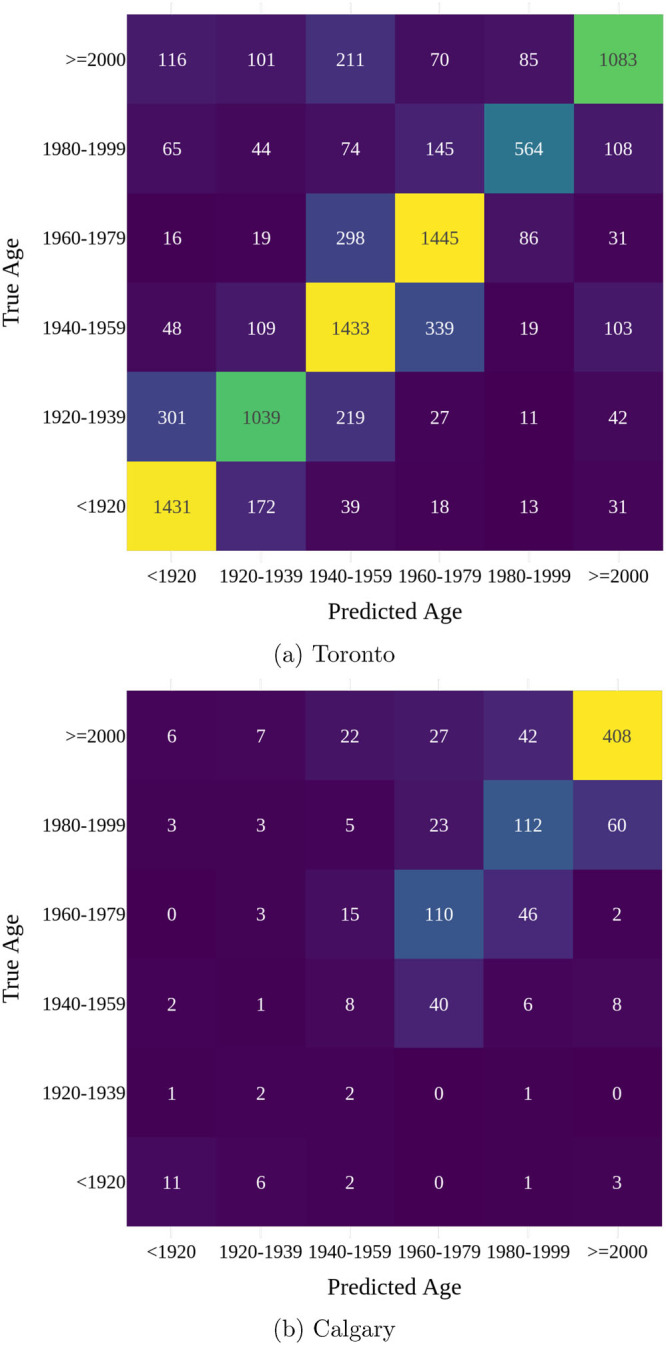
Confusion matrices of building age prediction for Toronto and Calgary. Underlying data are available in Table 4a and 4b of supporting information, respectively.

The prediction accuracy for the Toronto test set is high for all six age classes, as indicated by the balanced accuracy of $69.15\%$. For most age classes, most mispredictions are with adjacent time periods (e.g., predicting a pre-1920 building to be from 1920–1940), leading to a high value of $88.97\%$ for the off-by-one accuracy metric. A confusion matrix showing the prediction accuracy per age class is provided in Figure 4a. This matrix illustrates that most errors occur between adjacent age classes, which is consistent with the high off-by-one accuracy score observed.

#### Model performance in other cities

The model's performance varies significantly across the cities we considered. While the model achieves a MAE of 0.54 and an accuracy of over 70% for Toronto, the accuracy ranges between 40% and 65% for the five other cities. This variance could be attributed to factors such as the diversity in building styles, the age distribution of buildings, or the size and quality of the datasets available for each city. The one exception is Calgary, where the model has an accuracy of 65.89%. Inspecting the confusion matrix for Calgary in Figure 4b, we see that the vast majority of buildings in the dataset are fairly new (echoing Calgary's built stock which was predominantly built post-1958), which the model successfully classifies. However, the performance is much worse for other periods as captured in Calgary's balanced accuracy metric in Table 3.

### Area prediction

The area prediction results for each city are summarized on the right in Table 3. For Toronto, Calgary, and Hamilton, the model estimated building area with an MAPE of less than 20%—for example, for a 2000-square-foot building, this would indicate a range of estimates between 1600 and 2400 square feet. For Moncton, Montreal, and Victoria, the MAPE was worse, with Victoria recording the highest error. Notably, performance on this task is not directly linked to performance on the age task. For example, Hamilton was among the worst-performing cities in terms of MAE on the age task. However, the MAPE for Hamilton is nearly the same as for Toronto. This suggests that distinct factors influence the model's abilities to estimate building age and area. This discrepancy may be attributed to the nature of the data used for each task or to inherent differences in the model's ability to interpret and learn from different types of urban features.

Moreover, this observation underscores the importance of considering the specific characteristics of each city when developing predictive models. It is possible that factors such as historical development patterns, building renovations, or even the quality of the data sources available for each city could significantly impact the model's performance on different tasks. For example, the historical architecture in Hamilton might pose a challenge for accurate age estimation, while the layout and size of buildings could be more uniformly captured, leading to better area predictions.

Additionally, this finding highlights the potential need for task-specific model tuning or even the development of separate models optimized for different aspects of urban analysis. It emphasizes the complexity of urban environments and the challenges in creating a one-size-fits-all model for urban analytics tasks.

## DISCUSSION

Our technique is novel in that it not only predicts the age of buildings—an externally recognizable feature—but also predicts the internal floor area, which cannot be directly seen from the outside. The fact that our deep learning method achieves even reasonable accuracy in this task is quite exciting. This capability underscores the potential of deep learning to infer hidden attributes of buildings from visual data, expanding the scope of what can be analyzed and predicted using SVI. This dual capability of our model opens up new possibilities for urban analysis and planning, enabling a more comprehensive understanding of building stock and its characteristics.

Additionally, our approach uses a single street-view image for predictions, which is much simpler and more efficient than the patch analysis methods used by others. By utilizing one-shot analysis, our method significantly reduces computational complexity and processing time, making it easier to implement and scale for large datasets. This simplicity and efficiency further highlight the practical potential of our technique for real-world applications.

Furthermore, we analyzed the performance of our model by dividing the predicted floor areas into five bins of equal size. The MAPE for each bin shows consistent performance across different ranges of floor areas, with MAPE values generally around 15% –25%. While the error is slightly higher at the extremes (smaller and larger floor areas), it remains within a reasonable range and never dramatically deviates from the overall error. This consistency demonstrates the robustness and reliability of our approach across various building sizes, validating its applicability for real-world scenarios.

### Performance analysis

Our models' performance demonstrates that it is possible to accurately predict both the age and area of buildings from GSV images. Our hypothesis that external imagery is sufficient for an estimate of internal measurements is supported by the results of our modeling—while these estimates are not perfectly accurate, they clearly track with the true measurements. However, the generalization of a model trained on data from a single city to other cities is challenging for the approach we have proposed. This limited generalization could be explained by several factors. For instance, architectural styles and the distribution of building ages differ significantly between cities. Toronto's building stock may be more uniform in age or style compared to cities like Montreal, which has a wider variety of architectural eras. This diversity may challenge the model, which has been trained exclusively on Toronto data.

Many of the images in our dataset include foreground occlusions, such as cars, trees, or other obstacles. Early in the development of our model, we sought to investigate whether performance would be improved by filtering images from our dataset if they were too obstructed. This was accomplished by using a semantic segmentation model to predict the subject of each part of the image: building, road, sky, etc. We removed images where less than 10% of pixels were predicted to be directly of the building. While this may seem low, consider that information such as the sky and street outside a building are essential to determine the outline of the structure, and so a very high percentage is similarly undesirable. We found that this approach had only minor impact on performance while making the model more complicated and slower: for area prediction, it improved the MAPE from 23.39% to 22.27% at that stage of development, while for the age classification our accuracy improved only slightly from 61.03% to 63.65%. Due to these preliminary results we did not continue to include the semantic segmentation approach in our final model, and instead use all images.

For area prediction, the MAPE in Toronto, Calgary, and Hamilton is below 20%, indicating reasonable accuracy. However, the error rates for Moncton, Montreal, and Victoria are higher. This discrepancy could be due to the differences in building sizes and layouts in these cities or the limitations of GSV images in accurately capturing the full extent of a building's area.

**FIGURE 5:**
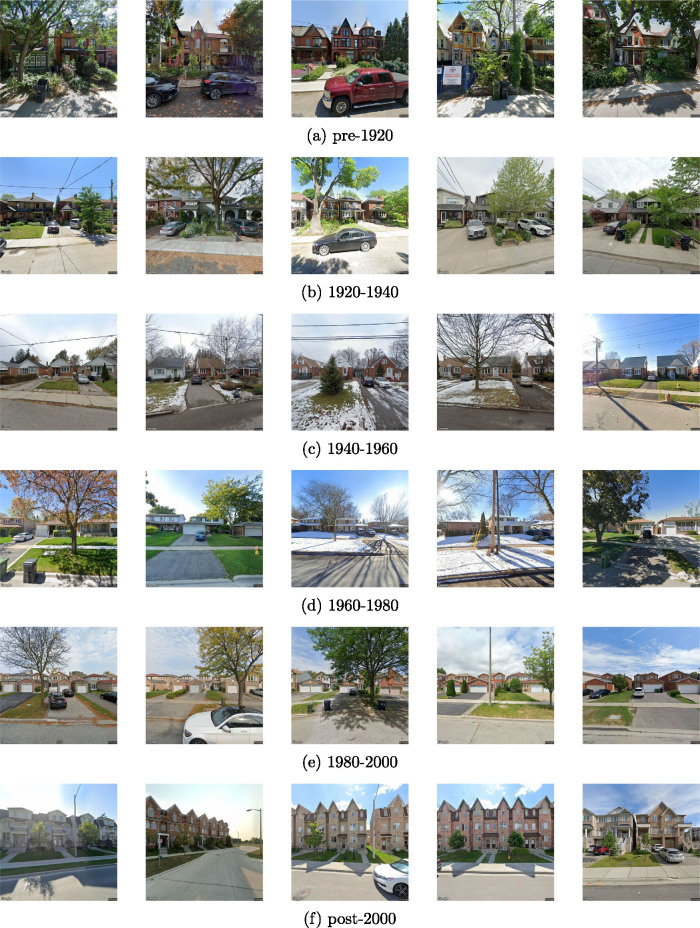
Top five correct age predictions for each age class.

To better understand the age prediction models and verify that our model learned to produce sensible predictions, we examine test set images that are correctly classified. Figure 5 shows the five houses of each age class that were correctly classified by the model with the highest probability scores. The trained model does capture the characteristics of houses built in different age classes. For example, houses built before 1920 typically have very high roofs and have no garage or driveway in the front yard. Houses built in 1940–1960 tend to have a longer driveway than houses built in 1920–1940. Houses with garages became more common after 1960. Before 2000, the number of storeys typically does not exceed two.

**FIGURE 6:**
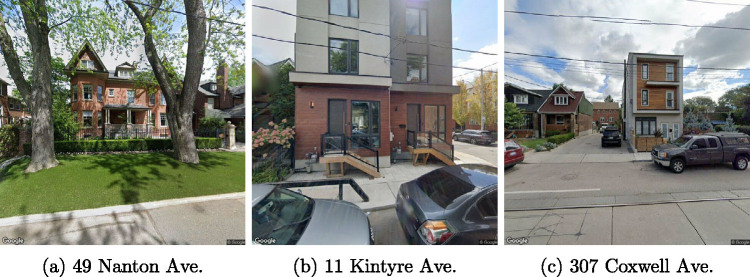
Buildings built post-2000 that were predicted as having been built pre-1920.

While the age prediction model worked quite well overall, some misclassifications were surprising, particularly the type of buildings that are most gravely misclassified: ones that were built post-2000 but predicted to have been built pre-1960. Figure 6 shows three such cases.

We find that some houses that are misclassified as being built in older classes exhibit the architecture style of older age classes. For example, Figure 6a shows a house located at 49 Nanton Avenue which was built in 2004 according to the RPS data. It exhibits an older architectural style typical in pre World War Two Toronto homes, and it is predicted as having been built between 1920 and 1940 by the trained model. We hypothesized that this confusion could be associated with a renovation to the house. While not described anywhere in the original data there is a fundamental question about at one point a thorough renovation (e.g., to the studs) constitutes a “new” building in the RPS data. We were able to confirm this hypothesis for 49 Nanton Avenue through a newspaper article discussing the renovation and later sale of the house. The article describes the house as a century old and extensively renovated including an addition. Early 20th-century details were “maintained or replicated” (e.g., the central stair) with classic features “added” (such as moldings and baseboards) (Yu, 2008). We hypothesize that this type of confusion occurs in many misclassified post-2000 homes as renovations (including the internal gutting of homes) are widespread in Toronto. This confusion both in terms of the labeling in the original dataset and the classification by the model highlights the complex nature of renovated buildings in stock assessment and related fields. They are ultimately a chimera that defies strict classification. They are also more the rule than the exception as renovations are a standard part of a decades-long building lifetime. This is an area for further research.

There are also cases when the style of the house is modern, but the model incorrectly predicts that it has been built much earlier (Figure 6b). The nature of this confusion requires further research.

In addition, we hypothesize that sometimes the model makes an incorrect prediction because it fails to identify the targeted house in the image. For example, in Figure 6c, the targeted house (307 Coxwell Ave) is the one that is behind the lane between the two houses that are closer to the camera. However, there is no easy way to avoid this automatically, and it would require manual manipulation of the images obtained from GSV or computer vision to identify house numbers (when visible).

### Comparison to prior work

No existing work predicts the area of buildings from a single street-view image so we cannot compare our accuracy to existing literature for the area task. For age prediction, we find it hard to directly compare the performance of our model to the accuracies reported in the literature due to vast differences in location, number of age classes, range of each class, and the source of the input images of each study. Compared to existing studies, the images we use as the input are noisy. For example, there are cases where there is more than one house in each image or where the house of interest is occluded. Despite this, our approach achieves comparable accuracy to existing methods on the age task, and state-of-the-art accuracy on the area task. Our results show the feasibility of estimating building attributes from street-view images and the potential for large-scale analysis of the built environment, even with image data that is not well curated, which would be the case for any neighborhood or city-scale use of building images. Automatic assessment of building area and age thus has the potential to provide a basis for bottom-up material stock analysis at city scale.

### Potential ideas for improved models

Our results show the potential to use ML to predict building attributes from noisy data and fairly straightforward inputs (single street-view images generated from address data). We tried more complicated approaches to see if that would improve performance with no meaningful return such as using multiple images per building, adding aerial building, extracting the building pixels only (not including background pixels in training or prediction). In future work, in addition to image-based data, building footprint data could be added where available, giving firm data on building maximum depth and helping to differentiate the target building from adjacent ones. Such an approach, however, relies on accurate footprint data, which is only occasionally available and would make the process of analysis more involved compared to the single-shot method presented here.

### Conclusion

Our study underscores the significant potential of applying machine learning techniques to better understand the built environment. By leveraging GSV images, we have demonstrated the feasibility of accurately predicting both the age and internal floor area of buildings—two critical attributes for urban metabolism, material flow analysis, and embodied GHG assessment.

The primary findings of our research are twofold: first, our models achieved state-of-the-art accuracy in predicting building area, a task that has not been previously attempted using single street-view images. Second, the ability to predict internal floor area from external images is particularly noteworthy, as it highlights the power of deep learning to infer hidden building attributes, thus opening new avenues for urban analysis and planning.

Our results also reveal the complexities and challenges of generalizing models across diverse urban landscapes. Differences in architectural styles and the distribution of building ages across cities pose significant challenges to model generalization. Despite these challenges, our approach has shown that with appropriate adjustments and enhancements, machine learning can be a robust tool for large-scale analysis of the built environment.

Ultimately, leveraging advanced AI tools for the assessment of the built environment aligns with the urgent need for innovative solutions in the face of global climate challenges. By providing a basis for bottom-up material stock analysis at the city scale, our study contributes significantly to sustainable development. The insights gained from our work can inform policy and decision-making aimed at reducing GHG emissions and promoting climate resilience.

In summary, our research not only advances the field of urban studies but also demonstrates the practical applications of AI in addressing some of the most pressing global issues. The novelty and implications of our findings underscore the importance of continued exploration and refinement of AI methodologies for sustainable urban development.

## Supplementary Information

The supporting information spreadsheet contains specific counts used in the histograms of Figure 1 and the heatmaps of Figure 4. Figure 1a presents a histogram of living area values, while Figure 1b shows a histogram of year built values. Figure 4a includes a heatmap of age prediction task results for Toronto, and Figure 4b provides a heatmap for Calgary.

## Data Availability

The data that support the findings of this study are available from various third parties. Restrictions apply to the availability of these data, which were used under license for this study. Cityscapes Dataset: This dataset is publicly available from the creators of the Cityscapes project. Interested researchers can access the dataset at www.cityscapes-dataset.com with permission from the Cityscapes project team. Real Property Solutions (RPS) Property Valuation Dataset: The RPS dataset was used under an agreement with Real Property Solutions (RPS). This data is not publicly accessible, available only upon direct request to Real Property Solutions (RPS), and subject to their data-sharing policies. Google Street View Images: The images from Google Street View are readily accessible and can be obtained through the Google Street View service. However, these images were used under specific terms of service and are not available from the authors of this study. Researchers should refer to Google's policies for data access and usage. Researchers interested in the datasets used in this study are advised to contact the respective third parties directly to obtain access in accordance with their terms and conditions.
